# A case of left adrenal echinococcosis misdiagnosed as a left renal cyst: Case report

**DOI:** 10.1097/MD.0000000000047934

**Published:** 2026-03-13

**Authors:** Xin Guo, Chang-Xing Ke, Ruo-Liu Xiao, Dong-Lin He, Zhi-Jing Wang, Zhi-Yuan Zhang, Yong Yang, Jian-Lin Yang

**Affiliations:** a Department of Urology, The Second Affiliated Hospital of Kunming Medical University, Kunming, China; b Department of Urology, The Puer People’s Hospital, Kunming, China.

**Keywords:** adrenal glands, case report, diagnosis, echinococcosis, treatment

## Abstract

**Rationale:**

Echinococcosis is a zoonotic parasitic disease caused by Echinococcus larvae, with high prevalence of cystic echinococcosis and alveolar echinococcosis forms in western China. Clinical manifestations depend on cyst location, size, and complications. It is associated with animal husbandry in endemic regions and is considered an occupational disease in exposed populations.

**Patient concerns:**

A 10-year-old patient was admitted following imaging identification of a left renal cystic lesion. Computed tomography revealed a 6.5 cm × 5.5 cm non-enhancing cystic mass in the left subphrenic region and hepatorenal space. Laboratory results: complete blood count, biochemistry, hormonal assays, and urinalysis – were within normal limits. Based on age and clinical presentation, a preliminary diagnosis of simple renal cyst was made.

**Diagnoses:**

The patient underwent laparoscopic partial cystectomy. Intraoperatively, the cyst was found to originate from the adrenal gland. Histopathological and immunohistochemical examination confirmed adrenal echinococcosis.

**Interventions:**

The patient underwent laparoscopic partial cystectomy.

**Outcomes:**

The cyst originated from the adrenal gland. Histopathological and immunohistochemical examinations confirmed it as adrenal echinococcosis.

**Lessons:**

When diagnosing renal cystic lesions, do not conclude hastily from routine imaging and prelims. Consider rare causes like adrenal echinococcosis. Do serological tests, stress differential diagnosis to avoid misdiagnosis.

## 1. Introduction

Renal cysts are common benign renal lesions, usually presenting as single or multiple cystic masses. These cysts vary in size, and most patients are asymptomatic. However, when the cysts enlarge, they may compress adjacent tissues and cause complications such as low back pain, abdominal discomfort, palpable masses, or hematuria.^[1]^ Echinococcosis is a zoonotic disease caused by infection with Echinococcus tapeworm larvae in humans. It is more prevalent in western China, where cystic echinococcosis and alveolar echinococcosis are more common.^[2]^ The clinical manifestations of this disease depend on the location and size of the cysts and the presence of complications. For a long time, echinococcosis has been considered a zoonotic parasitic disease, referred to as a disease of animal origin. In recent years, epidemiological investigations have shown that it is mainly related to animal husbandry and exhibits geographic endemicity. It is characterized by occupational hazards in endemic areas and has been classified as an occupational disease in certain groups of people.^[3]^ When echinococcosis infects the kidneys or adrenal gland, it presents as a lesion in these organs. On computed tomography (CT) or contrast-enhanced CT, it appears similar to renal cysts. We report the case of a 10-year-old patient who was preoperatively misdiagnosed with a left renal cyst; however, intraoperative exploration revealed that the lesion was located in the left adrenal gland. This case highlights the importance of thorough differential diagnosis in renal cystic diseases and the need to consider rare conditions to optimize treatment efficacy and reduce patient suffering.

## 2. Case description

**Figure 1.**
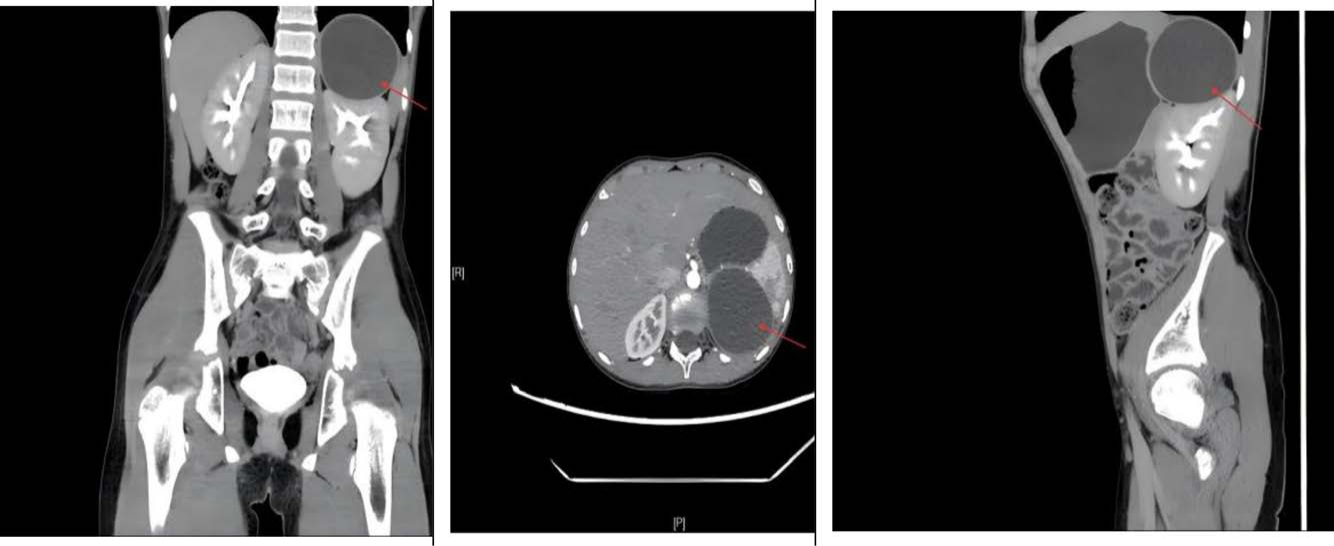
A well-defined cystic mass, approximately 6.5 cm × 5.5 cm in size, was seen under the left diaphragm. The area indicated by the red arrow is the cystic mass.

**Figure 2.**
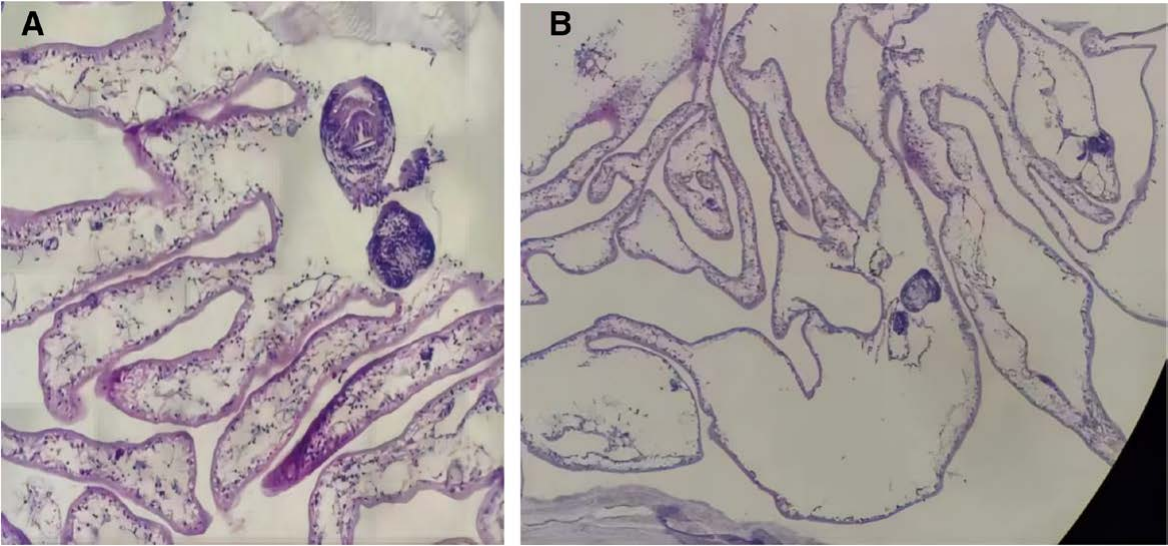
(A) cyst wall: small glandular (adenoid-like) structures and tissues rich in blood sinuses were observed locally within the simple cyst; these structures appeared consistent with parasitic worm tissue. (B) cyst contents: consisted of pink lamellar material that resembled parasitic worm tissue.

**Figure 3.**
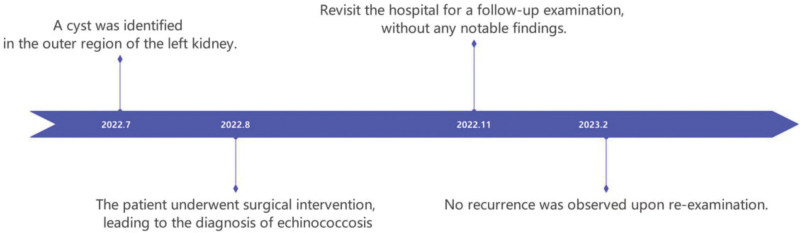
Clinical timeline of left renal echinococcosis patient.

A 10-year-old patient was found to have a cystic mass in the space between the liver and kidney during a CT examination for an upper respiratory tract infection 1 month ago. The patient did not have accompanying symptoms such as hematuria and abdominal pain and was then transferred to our hospital for further examination and treatment. At our hospital, a complete physical examination, complete blood cell count, biochemistry test, hormone test, and urinalysis were all normal. The enhanced CT scan showed a cystic mass of about 6.5 cm × 5.5 cm in size under the left diaphragm, with a clear boundary, low-density shadow, homogeneous density, and no obvious enhancement (Fig. 1). Furthermore, the rest of the examination did not show any special features. The mother complained on behalf of the child that they had lived in the area for a long time. She stated that our area was not an endemic area for echinococcosis and that there was no history of contact with infected water. She also mentioned that the child had not been vaccinated on time, had not visited pastoral or epidemiologic areas, and had not had a systematic physical examination since birth. Renal cysts can be divided into 4 types according to the Bosniak classification: simple (Bosniak type I and II) and complex (Bosniak type III and IV).^[4]^ The characteristics of each type are shown in Table 1. Based on the patient’s examination and clinical manifestations, we diagnosed the patient with a simple renal cyst in the left kidney before surgery and chose the surgical plan of laparoscopic retroperitoneal cystectomy. During the surgical treatment, when we separated the tissues, we found that the source of the cyst was not the kidney but the adrenal gland. Therefore, we took the approach of cyst stripping. The cyst was completely stripped and removed from the body. A small amount of turbid cystic fluid flowed out after incision of the cyst wall, and a jellylike inner capsule was seen inside the outer capsule. Postoperative pathological examination results were as follows: the cyst wall showed a simple cyst localized with a few small adenoidal structures and tissues rich in blood sinuses; parasitic worm tissue was suspected. The cyst contents were sent for examination as powder-stained flat material, and parasitic worm tissue was also suspected (Fig. 2), which was consistent with hydatid disease (echinococcosis) caused by Echinococcus granulosus. Although the initial misdiagnosis could have increased the risk of intraoperative cyst fluid spillage and postoperative recurrence, the patient had a good recovery and showed no recurrence during the 6-month follow-up after discharge, below is the clinical visit timeline of the patient (Fig. 3).

**Table 1:** Bosniak renal cyst classification system: an updated version.

Category characteristics	
I	Hairline-thin wall; water attenuation; no septa, calcifications, or solid components; nonenhancing
II	Two types: 1. Few thin septa with or without perceived (not measurable) enhancement, fine calcification or a short segment of slightly thickened calcification in the wall or septa. 2. Homogeneously high – attenuating masses ≤3 cm that are sharply margined and do not enhance
II F	Two types: 1. Minimally thickened or more than a few thin septa with or without perceived (not measurable) enhancement that may have thick or nodular calcification. 2. Intra renal nonenhancing hyperattenuating renal masses <3 cm
III	Thickened or irregular walls or septa with measurable enhancement
IV	Soft tissue components (i.e., nodule[s]) with measurable enhancement

Adapted from Ref^[4]^.

## 3. Diagnostic assessment

The cyst measured 6.5 cm × 5.5 cm, and its contents appeared as a transparent, jellylike substance. Postoperative examination confirmed that the cyst consisted of a cyst wall and cystic fluid. Pathological analysis of the cyst wall revealed simple cysts localized within a few small adenoidal structures and tissues rich in blood sinuses, which contained parasitic larval elements. The cystic fluid was examined and identified as a stained, platelike material consistent with parasitic tissue. Immunohistochemical staining showed cytokeratin-P (epithelium positive), synaptophysin (positive), melan A (positive), chromogranin A (negative), Ki-67 (<1% positive), and androgen receptor (negative). Based on these findings, the diagnosis was revised from a simple renal cyst of the left kidney to left adrenal echinococcosis, consistent with the anatomical location of the left adrenal gland. Following the pathological diagnosis, the patient received postoperative albendazole treatment for 3 months. At 6 months postoperatively, no recurrence was observed, and the patient continues to be followed up.

## 4. Discussion

In a 2024 case report by Tajmalzai, an 18-year-old patient with adrenal hydatidosis was diagnosed preoperatively after imaging raised suspicion, which was subsequently confirmed by serology. Albendazole was initiated 2 weeks before surgery. Then, a transperitoneal right adrenalectomy was performed to reduce the risk of recurrence.^[5]^ The patient exhibited no obvious clinical signs and was not from an endemic area; therefore, adrenal hydatidosis was not initially suspected. Routine laboratory tests were nonspecific, leading to a preoperative misdiagnosis of a renal cyst.^[6]^ Most patients with renal cysts did not have obvious clinical manifestations, although some presented with symptoms such as pain, hematuria, or urinary tract obstruction.^[7]^ In this case, the presentation was consistent with that of a simple cyst, characterized by a hairline-thin wall, water attenuation, absence of septa, calcifications, or solid components, and no significant enhancement on enhanced CT.^[4]^

The symptoms of echinococcosis vary mainly as localized symptoms, which are chiefly due to the growth location, size, and various effects of Echinococcus larvae on the surrounding tissues.^[8]^ For example, in the liver, there may be nausea, vomiting, and liver cysts. Jaundice may occur if the cyst is located near the bile ducts.^[9]^ In the lungs, there may be respiratory symptoms such as shortness of breath and chest pain.^[10]^ Involvement of the cranium may lead to epileptic seizures, headache, vomiting, and other symptoms of elevated intracranial pressure.^[11]^ Parasitic involvement of the bone can easily cause fractures. Based on this case, the patient’s history, symptoms, physical examination, and test results were similar to those of renal cysts. The diagnosis of a renal cyst was mainly based on imaging. Moreover, the patient had no history of contact with an infected area or living abroad, which is uncharacteristic in this case. Because we failed to consider the possibility of echinococcosis, the preoperative diagnosis was left renal cyst. Consequently, laparoscopic retroperitoneal cystectomy was chosen. The main treatment options for renal cysts are aspiration sclerotherapy and surgery.^[7]^

However, we chose laparoscopic retroperitoneal cystectomy via the retroperitoneal approach, considering the patient’s age and the possibility of recurrence. In this case, we did not consider the possibility of echinococcosis. A previous study reported that the sensitivity (100%) and specificity (96%) of serologic immunoassay were very helpful for the early diagnosis of echinococcosis.^[12]^ Retrospectively, if we had performed preoperative serologic testing and detected antibodies to the parasite, we could have improved the accuracy of our preoperative diagnosis. With a clear diagnosis, selecting the appropriate treatment strategy may reduce the recurrence of echinococcosis. Recurrence of echinococcosis occurs in some patients during clinical treatment, and most of the reasons for it are mainly related to the choice of surgical procedures. Intraoperative spillage of cystic fluid due to preoperative misdiagnosis is the main cause of recurrence.

Complete exocystectomy is considered the ideal surgical procedure. In this procedure, the cystic fluid is first removed with a fine needle to prevent its spillage. Then, the internal capsule is removed. The internal capsule is only mildly adherent to the external capsule, making it easy to be peeled off and often allowing it to be removed intact. This procedure should be performed on Echinococcus larvae in the lungs, brain, and bones.^[13]^ The prognosis of this disease is good, and distant metastases are rare, although some patients may experience multiple localized recurrences. Therefore, all patients should be followed up regularly for a long period of time. Albendazole and mebendazole are effective in the treatment of echinococcosis and are the preferred treatment options for inoperable cases or for reducing the recurrence of postoperative echinococcosis.^[14]^ In the case we reported, the lack of a clear preoperative diagnosis increased the likelihood of postoperative recurrence, possibly due to inadequate initial management. Based on the pathologic findings, we chose albendazole treatment. During the 6-month follow-up after the operation, no recurrence was observed.

## 5. Conclusion

In the case we reported, a preoperative misdiagnosis of a left renal cyst – when the actual condition was echinococcosis of the left adrenal gland – led us to implement an inappropriate treatment plan for the patient. This inappropriate treatment aggravated the patient’s condition and reduced the likelihood of a successful outcome. This case serves as a reminder to clinicians to be vigilant in diagnosing simple cysts of the kidney. Furthermore, preoperative serologic tests can enhance diagnostic accuracy and assist in selecting appropriate treatment options.

## Author contributions

**Resources:** Xin Guo, Zhi-Jing Wang.

**Supervision:** Chang-Xing Ke, Ruo-Liu Xiao.

**Validation:** Xin Guo, Ruo-Liu Xiao.

**Writing – original draft:** Xin Guo, Dong-Lin He, Zhi-Jing Wang.

**Writing – review & editing:** Chang-Xing Ke, Zhi-Yuan Zhang, Yong Yang, Jian-Lin Yang.
